# Preventive effect of small molecule active substances on septic cardiomyopathy after abdominal trauma: a systematic review and meta-analysis

**DOI:** 10.3389/fphar.2025.1665372

**Published:** 2026-01-06

**Authors:** Gen Ouyang, Neng Wang, Yujuan Liu, Chuang Yang, Peng Zeng, Tao Gong, Lu Tao, Ying Zheng, Guiying Ye, Hong Li, Chi Che, Longhai Wang, Nai Zhang

**Affiliations:** 1 Department of General Surgery, Jiangxi Province Hospital of Integrated Chinese and Western Medicine, Nanchang, Jiangxi, China; 2 Graduate School, Jiangxi University of Chinese Medicine, Nanchang, Jiangxi, China; 3 Department of Emergency, Jiangxi Province Hospital of Integrated Chinese and Western Medicine, Nanchang, Jiangxi, China; 4 Department of Critical Care Medicine, Jiangxi Province Hospital of Integrated Chinese and Western Medicine, Nanchang, Jiangxi, China

**Keywords:** septic cardiomyopathy, abdominal sepsis, abdominal trauma, small molecules, meta-analysis

## Abstract

**Objective:**

This systematic review and meta-analysis aimed to comprehensively evaluate the preventive effects and mechanisms of active small molecules against septic cardiomyopathy (SCM) induced by abdominal trauma or abdominal-origin sepsis in animal models.

**Methods:**

The PubMed, Embase, Web of Science, Scopus and Cochrane Library databases were searched in January 2000- May 2025 for studies assessing active small molecules in animal SCM models. The standardised mean difference ([SMD], Hedges’ g) with 95% confidence intervals (CIs) was calculated using random-effects meta-analysis. Subgroup analyses were conducted according to molecular categories, sepsis induction methods, and outcome measures. Methodological quality was assessed using the Systematic Review of Laboratory Animal Experiments risk-of-bias tool.

**Results:**

Seventeen studies met the inclusion criteria. The pooled meta-analysis showed that active small molecules had an overall beneficial but heterogeneous effect on SCM (SMD = 1.47, 95% confidence interval [CI]: −0.52–3.47, I^2^ = 92.9%). Subgroup analyses revealed large but imprecise effects for polyphenols (SMD = 4.81, 95% CI: −5.25–14.87) and neutral effects for flavonoids (SMD = −0.45, 95% CI: −3.64–2.73). The efficacy was greater in lipopolysaccharide-induced sepsis models (SMD = 2.86) compared with cecal ligation and puncture models (SMD = 0.16). Outcomes involving cardiac injury biomarkers (creatine kinase and creatine kinase isozymes) showed consistently positive and robust effects, while functional outcomes (e.g., left ventricular ejection fraction) exhibited inconsistent results. Sensitivity analyses confirmed robustness, while funnel plots indicated possible publication bias (Egger’s test, p = 0.123). Methodological limitations, including incomplete reporting of randomisation, blinding and allocation concealment, were commonly observed.

**Conclusion:**

Active small molecules demonstrate generally positive yet heterogeneous preventive efficacy against SCM in animal models, with polyphenolic compounds in particular showing notable potential. Variability across molecular categories, sepsis models and measured outcomes highlights the need for standardised methodologies in future studies.

## 1 Introduction

Sepsis is a life-threatening clinical syndrome characterised by a dysregulated host response to infection, resulting in extensive tissue damage, multi-organ dysfunction and high global mortality (Suzuki et al., 2025; Jinzhong Wang and Jian Fu, 2025). Among the various complications caused by sepsis, septic cardiomyopathy (SCM) is a particularly severe manifestation and a significant factor in morbidity and mortality in critically ill patients (Mohammad et al., 2022; Wang et al., 2024). The condition is defined as acute myocardial dysfunction associated with sepsis, typically manifested by reduced left ventricular ejection fraction (LVEF), impaired myocardial contractility and elevated cardiac biomarkers in the absence of coronary artery obstruction or direct myocardial injury (Yuan et al., 2019).

Abdominal trauma and subsequent abdominal sepsis are common clinical conditions leading to SCM, with unique pathophysiological mechanisms that exacerbate myocardial injury and dysfunction (Pan et al., 2024; Shen et al., 2024; Su et al., 2024). Mechanistically, abdominal sepsis induces systemic inflammation, characterised by significant elevations in proinflammatory cytokines (e.g., tumour necrosis factor-α [TNF-α], interleukin-6 [IL-6]), excessive oxidative stress, mitochondrial dysfunction and subsequent cardiac cell apoptosis and dysfunction (Latifi and Smiley, 2023; Adenuga and Adeyeye, 2023). Despite advances in sepsis management, including early antibiotic treatment, fluid resuscitation and vasoactive drug support, targeted therapies specifically targeting SCM remain limited, highlighting the urgent need for new effective preventive or therapeutic strategies.

In recent years, increasing preclinical evidence has shown that active small molecules, including polyphenols, flavonoids, saponins, antioxidants, glycosides and other bioactive compounds, may have significant cardioprotective potential against septic myocardial injury through multiple mechanisms, including anti-inflammatory effects, anti-oxidative stress response, mitochondrial protection and reduced cardiomyocyte apoptosis (Wang et al., 2021; Bayly-Jones et al., 2022; Lee et al., 2022). For example, resveratrol, a phenotypic polysaccharide, has been shown to have mitochondrial protective effects and the ability to significantly reduce cardiac injury biomarkers in rodent models of endotoxemia. Flavonoids such as quercetin and luteolin have also been reported to reduce cardiac dysfunction and inflammation in animal sepsis models, although their efficacy varies, depending on the experimental setting (Zhao et al., 2023). Despite preliminary evidence of the promising efficacy of these small molecules, a comprehensive quantitative review of their efficacy and mechanistic characteristics in animal models of SCM remains limited.

Abdominal trauma and subsequent intra-abdominal sepsis are major causes of critical illness in surgical and trauma populations and are associated with a high incidence of multiple organ dysfunction, including SCM (Sartelli et al., 2024; Mureşan et al., 2018). Abdominal-origin sepsis has pathophysiological characteristics such as ongoing peritoneal contamination, bacterial translocation and the potential for intra-abdominal hypertension/abdominal compartment physiology, which may produce distinct inflammatory and haemodynamic profiles compared with other sepsis sources (Mureşan et al., 2018; MacFie, 2004).

Accordingly, this systematic review and meta-analysis aimed to comprehensively evaluate the preventive effects of various active small molecules on SCM induced by abdominal trauma or abdominal sepsis in animal models. Specifically, we aimed to systematically evaluate the overall efficacy of these small molecules, identify potential sources of heterogeneity through subgroup analyses (e.g., molecular class, sepsis model, outcome measures), explore potential protective mechanisms and rigorously evaluate the methodological quality of different studies. We hypothesised that active small molecules could significantly prevent SCM through anti-inflammatory, antioxidant and mitochondrial protective mechanisms, although the extent of their effects varied, based on the specific molecular class and experimental model. Our findings aim to provide strong preclinical evidence to guide future translational research and clinical trials targeting SCM.

## 2 Methods

### 2.1 Protocol and registration

This systematic review and meta-analysis were conducted according to the Preferred Reporting Items for Systematic Reviews and Meta-Analyses (PRISMA) guidelines.

### 2.2 Search strategy

A systematic literature search was conducted in 5 electronic databases (time: January 2000- May 2025), regardless of language: PubMed, Embase, Web of Science, Scopus, and Cochrane Library. The search strategy included: a combination of relevant keywords and MeSH terms related to sepsis (‘sepsis’, ‘endotoxaemia’, ‘cecal ligation and puncture’, ‘CLP’), cardiomyopathy (‘cardiomyopathy’, ‘myocardial dysfunction’, ‘cardiac dysfunction’, ‘cardiac injury’) and active small molecules (e.g., ‘polyphenols’, ‘flavonoids’, ‘saponins’, ‘glycosides’, ‘antioxidants’, ‘phenylpropanoids’, ‘indolamines’).

### 2.3 Inclusion criteria

Studies that met all of the following inclusion criteria were included.

Subjects: Animal models (mice or rats) with intra-abdominal sepsis or traumatic sepsis.

Interventions: *In vivo* administration of active small molecules as preventive or therapeutic interventions for SCM.

Controls: The study included appropriate control groups (sepsis or model groups without active molecules).

Outcomes: Clear reporting of myocardial outcomes, including cardiac function measures (LVEF), mean arterial pressure (MAP), left ventricular developed pressure [LVDP]) and myocardial injury biomarkers (creatine kinase [CK], CK isozymes [CK-MB], adenosine triphosphate (ATP), cytochrome c oxidase [CcO]).

Study design: Controlled animal studies providing quantitative outcome data (mean, standard deviation [SD] or standard error and the number of animals per group).

Studies were excluded based on the following criteria: non-abdominal sepsis or irrelevant models; cellular or clinical studies lacking animal data; reviews, editorials, comments or conference abstracts; studies lacking clear quantitative outcome measures.

### 2.4 Study selection

**FIGURE 1:**
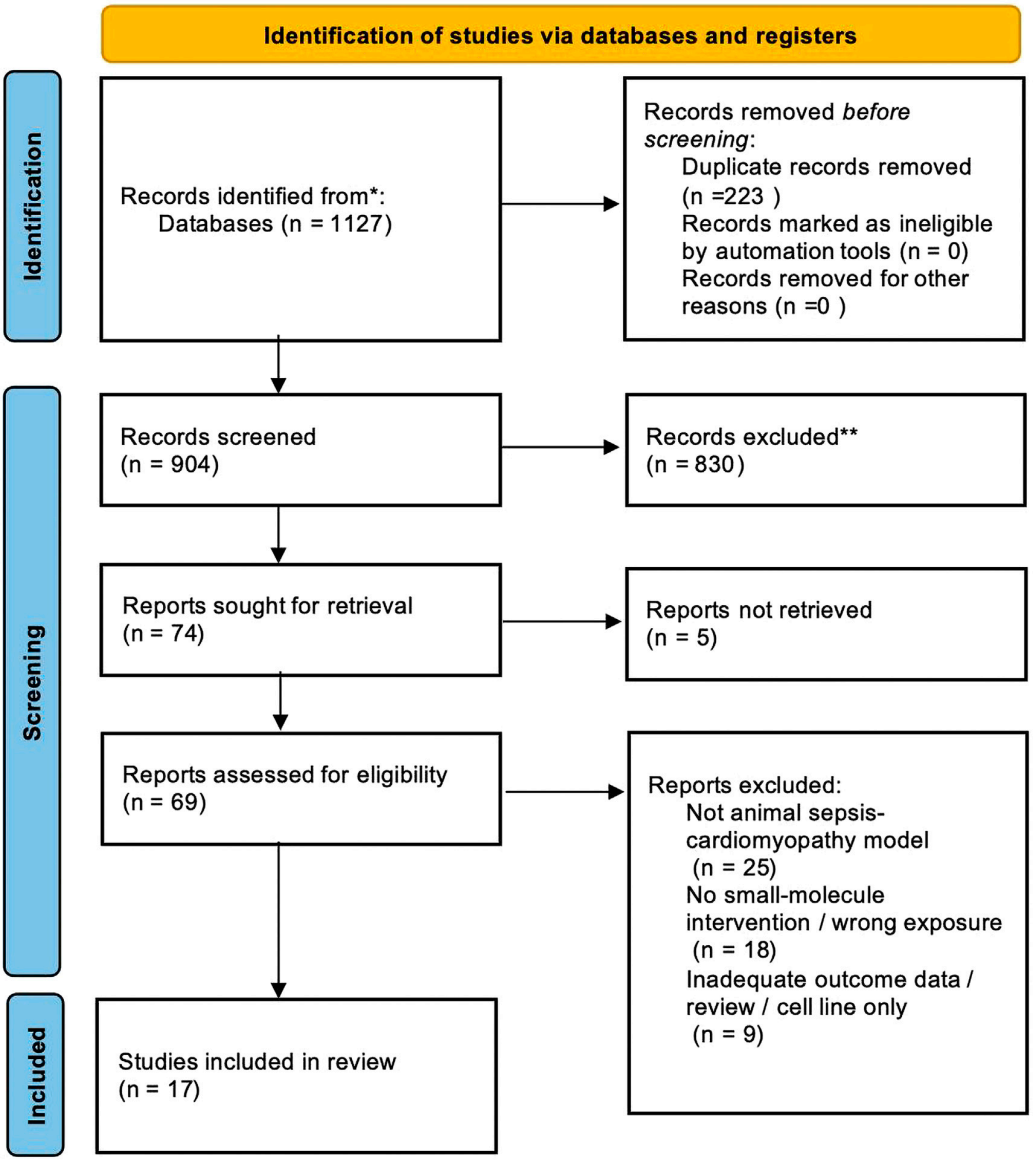
PRISMA 2020 flow diagram.

Two reviewers independently screened titles and abstracts from the search results against the pre-specified inclusion and exclusion criteria. Full texts were retrieved for records judged potentially eligible by either reviewer and were independently assessed by both reviewers. Discrepant judgments (<5%) at any stage were discussed and resolved by consensus; persistent disagreements were resolved by consulting a third reviewer. We documented the reasons for exclusion at full-text review. The complete selection process is presented in the PRISMA flow diagram (Figure 1).

### 2.5 Data extraction

Two reviewers independently extracted study-level data using a pre-piloted and standardised data extraction form. Extracted items included:

Study number: author and year of publication.

Animal characteristics: species, strain, age, and sex.

Sepsis model: lipopolysaccharide (LPS) dose, cecal ligation and puncture (CLP) procedure and trauma method.

Intervention details: name and molecular class of active molecule, dose (mg/kg), route of administration and time and frequency of administration.

Outcomes: cardiac function parameters (LVEF, MAP, LVDP), biochemical indices (CK, CK-MB, ATP, CCO), inflammatory markers (e.g., TNF-α, IL-6), oxidative stress markers (malondialdehyde(MAD), GPx4) and survival data.

Study design details: randomisation, blinding and sample size.

Discrepancies in extracted values were cross-checked and resolved by consensus; if a consensus could not be reached, a third reviewer adjudicated.

### 2.6 Risk of bias assessment

Two reviewers independently assessed methodological quality and risk of bias using the Systematic Review of Laboratory Animal Experiments (SYRCLE) risk of bias tool. The tool assesses 10 aspects as follows: (1) sequence generation, (2) baseline comparability, (3) allocation concealment, (4) randomisation housing, (5) caregiver blinding, (6) randomised outcome assessment, (7) assessor blinding, (8) incomplete outcome data, (9) selective outcome reporting and (10) other sources of bias. Each domain was judged as ‘low risk’, ‘unclear risk’ or ‘high risk’, based on the information reported in the article. To translate domain-level judgments into an overall study-level classification we applied a pre-specified conservative rule: (1) studies with any ‘high risk’ rating in 1 of 3 key domains (random sequence generation, allocation concealment or outcome assessor blinding) were classified as overall having high-risk; (2) if none of the key domains were ‘high’ but 2 or more domains were rated as ‘unclear’, the study was classified as overall having unclear risk; (3) studies with no ‘high’ domains and at most 1 ‘unclear’ domain were classified as overall having low risk. This approach prioritised the identification of studies with critical design/reporting weaknesses and reduced the chance of overestimating internal validity due to incomplete reporting.

### 2.7 Statistical analysis

Statistical analysis was performed using the metafor package in R software (version 4.3.2, R Foundation for Statistical Computing, Vienna, Austria). For continuous outcomes, effect sizes were calculated as SMD (Hedges’ g) and their 95% confidence intervals (CIs). Due to expected heterogeneity, we pre-selected a random-effects model using restricted maximum likelihood. Statistical heterogeneity between studies was quantified using Cochran’s Q test and Higgins’ I^2^ statistic (where I^2^ > 50% indicated significant heterogeneity). Subgroup analyses were performed based on molecular class, sepsis model (LPS, CLP, trauma + LPS), and outcome measures (cardiac function vs. biochemical indicators) to explore the sources of heterogeneity.

Publication bias was visually assessed using funnel plots and statistically tested using Egger’s regression test, with p-values <0.05 indicating significant bias. Sensitivity analyses included leave-one-out analysis to assess the robustness of the pooled effect.

## 3 Results

### 3.1 Literature search and study screening

The database search initially identified a total of 1,127 articles. After deduplication using EndNote X9 (Clarivate Analytics, Connecticut, United States), 223 duplicate records were removed, leaving 904 unique records. The titles and abstracts of these 904 articles were independently screened by two reviewers based on predefined inclusion and exclusion criteria as follows: (i) animal models of abdominal sepsis or abdominal trauma sepsis; (ii) interventions involving active small molecules; and (iii) clear reporting of myocardial outcomes, such as cardiac function or myocardial injury markers. Disagreements between reviewers (<5%) were resolved through discussion and consensus. After initial screening, 830 records were excluded due to irrelevant topics, leaving 74 articles eligible for full-text search. Of these, 5 reports were inaccessible despite multiple attempts to obtain the full text from institutional libraries and authors. Therefore, 69 full-text articles were evaluated in detail to determine their eligibility for the search. At this stage, 52 articles were excluded for the following reasons: 25 studies did not use SCM animal models, 18 studies lacked appropriate small molecule interventions or used irrelevant exposure conditions, and 9 studies were reviews, *in vitro* experiments only, or did not report relevant outcome data. Ultimately, 17 studies met all of the inclusion criteria and were included in the quantitative comprehensive analysis (meta-analysis). Figure 1 shows the PRISMA flow chart, detailing the study screening process.

### 3.2 Study characteristics

Table 1 (Jinzhong Wang and Jian Fu, 2025; Yuan et al., 2019; Zhao et al., 2023; Zeng et al., 2023; Huajie, 2015; Cui et al., 2024; He et al., 2015; Jing et al., 2016; Li et al., 2017; Qu and Meng-fei, 2020; Liu et al., 2021; Zhou et al., 2022; Peng et al., 2022; Bin et al., 2022; Zhang et al., 2019; Lin et al., 2023; Liu and Su, 2023) summarises the characteristics of the 17 included studies. Ten studies (59%) used rats and seven (41%) used mice (mainly C57BL/6). The most common sepsis model was cecal ligation and puncture (CLP, n = 10, 59%), followed by lipopolysaccharide (LPS)-induced endotoxemia (n = 6, 35%), with one study (6%) using a trauma + LPS model. The active small molecules investigated fell into six pharmacological classes: flavonoids (n = 9, including one isoflavone), polyphenols (n = 3), saponins (n = 2). phenylpropanoids (n = 1), glycosides (n = 1), and antioxidants (n = 1). Doses ranged from 0.2 mg/kg (luteolin) to 500 mg/kg (vitamin C), administered via intraperitoneal, oral, or intravenous routes. Primary outcomes included cardiac function indices (LVEF, MAP, LVDP) and biochemical injury markers (CK, CK-MB, ATP, CCO). Most studies (n = 9) reported LVEF, and six reported CK or CK-MB.

**TABLE 1:** Study characteristics.

Study ID	Year	Active molecule	Category	Species	Model	Dose (mg/kg)	Route	Measurement timepoint (h)	Primary outcomes
He et al. (2015)	2015	Salidroside	Glycoside	Rat	LPS	40	p.o	6	CK-MB
Huajie (2015)	2015	Resveratrol	Polyphenol	Rat	LPS	8	i.p	6	ATP, CCO
Jing et al. (2016)	2016	Quercetin	Flavonoid	Rat	Trauma + LPS	20	i.p	12	LVDP
Li et al. (2017)	2017	Apigenin	Flavonoid	Mouse	LPS	50	i.p	18	LVEF
Yuan et al. (2019)	2019	Polydatin	Phenylpropanoid	Rat	CLP	30	i.p	48	LVEF
Qu and Meng-fei. (2020)	2020	Astragaloside IV	Saponin	Mouse	LPS	80	i.p	8	LVEF
Liu et al. (2021)	2021	Quercetin	Flavonoid	Mouse	CLP	200	p.o	24	MAP
Zhou et al. (2022)	2022	Puerarin	Isoflavone	Rat	LPS	100	i.p	24	LVEF, CK-MB
Peng et al. (2022)	2022	Rosmarinic acid	Polyphenol	Mouse	LPS	100	p.o	12	LVEF
Bin et al. (2022)	2022	Luteolin	Flavonoid	Mouse	CLP	0.2	i.p	24	LVEF
Zhang et al. (2019)	2019	Melatonin	Indoleamine	Rat	LPS	30	i.p	8	LVEF,CK
Lin et al. (2023)	2023	Quercetin	Flavonoid	Rat	CLP	40	i.p	24	CK-MB
Zhao et al. (2023)	2023	Quercetin	Flavonoid	Rat	CLP	40	i.p	24	CK-MB
Liu and Su (2023)	2023	Luteolin	Flavonoid	Mouse	CLP	0.2	i.p	96	Survival, MAP
Zeng et al. (2023)	2023	Resveratrol	Polyphenol	Rat	CLP	50	i.p	24	LVEF
Cui et al. (2024)	2024	Vitamin C	Antioxidant	Rat	CLP	500	i.v	24	LVEF, CK
Jinzhong Wang and Jian Fu. (2025)	2025	Ginsenoside Rc	Saponin	Mouse	CLP	20	p.o	24	LVEF, CK-MB

### 3.3 Risk of bias assessment

**FIGURE 2:**
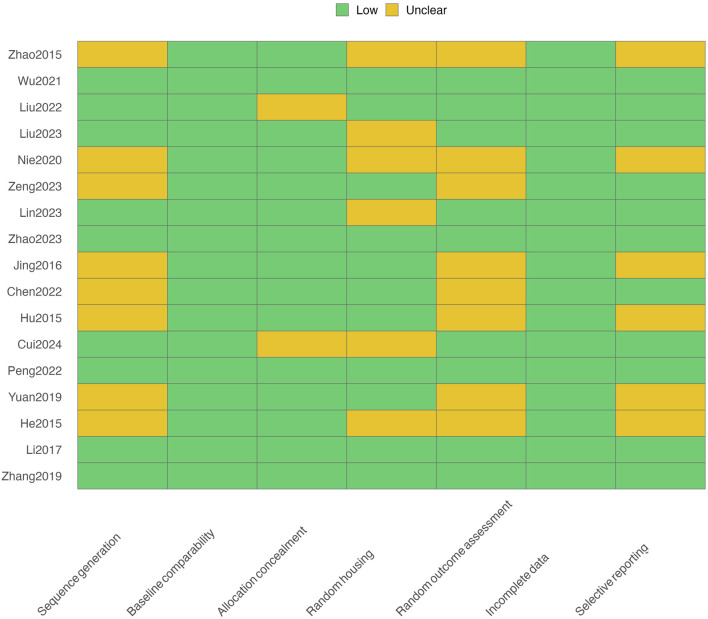
Risk of bias assessment.

Figure 2 presents the SYRCLE risk-of-bias assessment for the 17 included animal studies. Most items were rated as low risk, although several domains showed incomplete reporting.

Specifically, 9 studies (53%) clearly described adequate random sequence generation random sequence generation methods (e.g., random number tables or computer-generated lists), while 8 studies (47%) provided insufficient detail and were rated as “unclear risk.”

Baseline comparability was generally adequate across groups, with 100% of studies providing balanced characteristics.

For allocation concealment, 88% of studies (15/17) were rated low risk, while the remainder lacked clear information.

Random housing was adequately performed in 65% of studies (11/17), with 35% (6/17) rated as unclear.

Random outcome assessment was well reported in 9 studies (53%), though 8 studies (47%) lacked specific blinding details.

Incomplete outcome data were rarely an issue, with all studies (100%) fully reporting analyzed animals and exclusions.

Selective outcome reporting was low risk in 65% of studies (11/17), but 6 studies (35%) did not predefine outcomes or lacked accessible protocols.

Overall, the methodological quality was moderate, with the main limitations related to insufficient reporting of randomization and blinding procedures.

### 3.4 Quantitative synthesis

#### 3.4.1 Overall summary effect

**FIGURE 3:**
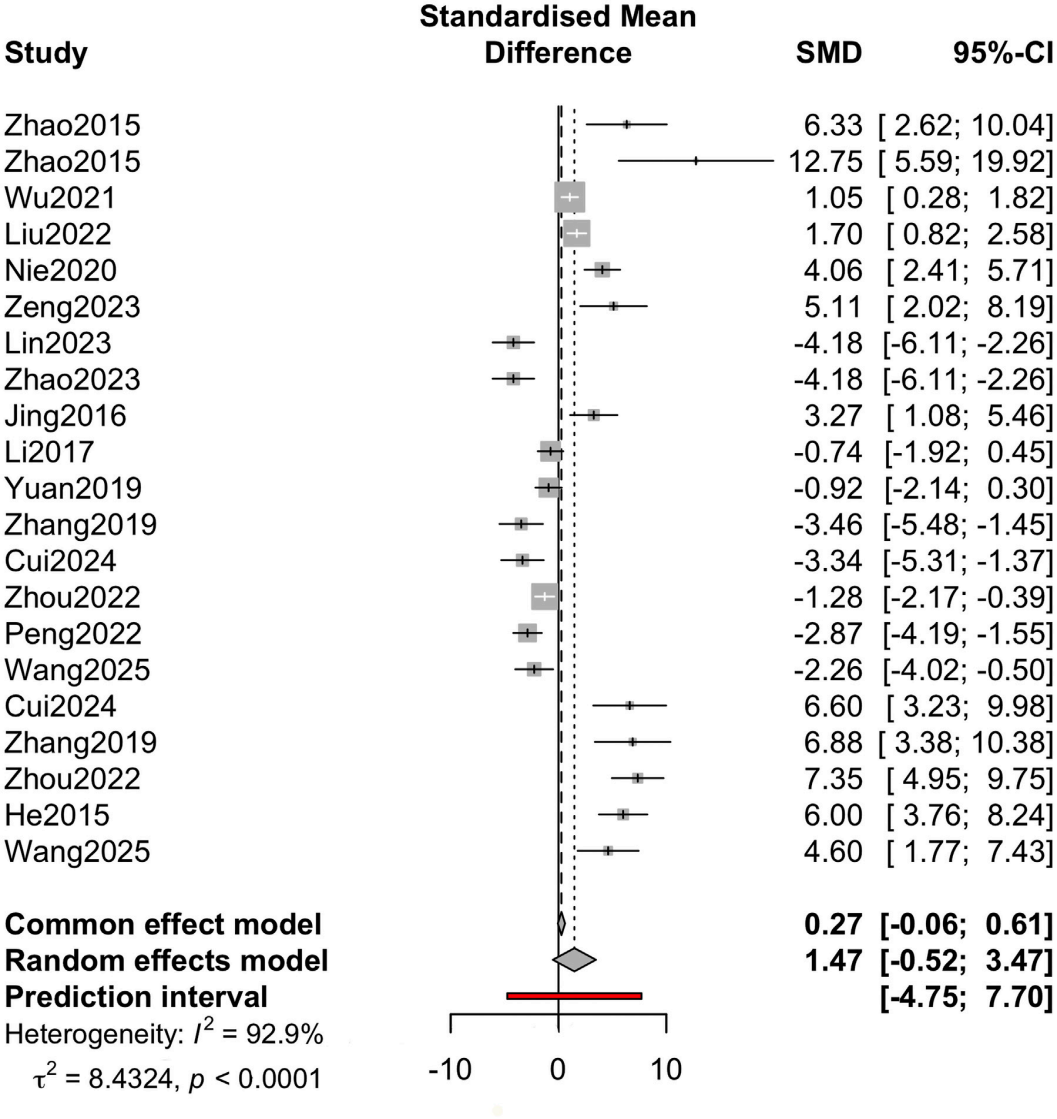
Overall summary effect Random-effects meta-analysis of all included studies showing standardized mean difference (Hedges’ g) and 95% confidence intervals (CIs). The model uses restricted maximum likelihood (REML). Heterogeneity statistics are reported as I^2^, τ^2^, and p-value for Cochran’s Q test. A 95% prediction interval is also shown to illustrate expected dispersion of true effects across similar studies. Individual study weights are indicated.

The overall summary effect of active small molecules on abdominal trauma or SCM was summarised using a random-effects meta-analysis. As shown in Figure 3, the SMD (Hedges’ g) across all studies was 1.47 (95% CI: −0.52–3.47), indicating a potential beneficial effect, although the confidence interval exceeded 0. There was significant statistical heterogeneity among the studies (I^2^ = 92.9%, p < 0.0001), indicating that the results were significantly different among the included studies. The wide prediction interval (−4.75–7.70) indicated that the true effect was significantly different between the studies.

#### 3.4.2 Subgroup analysis

**FIGURE 4:**
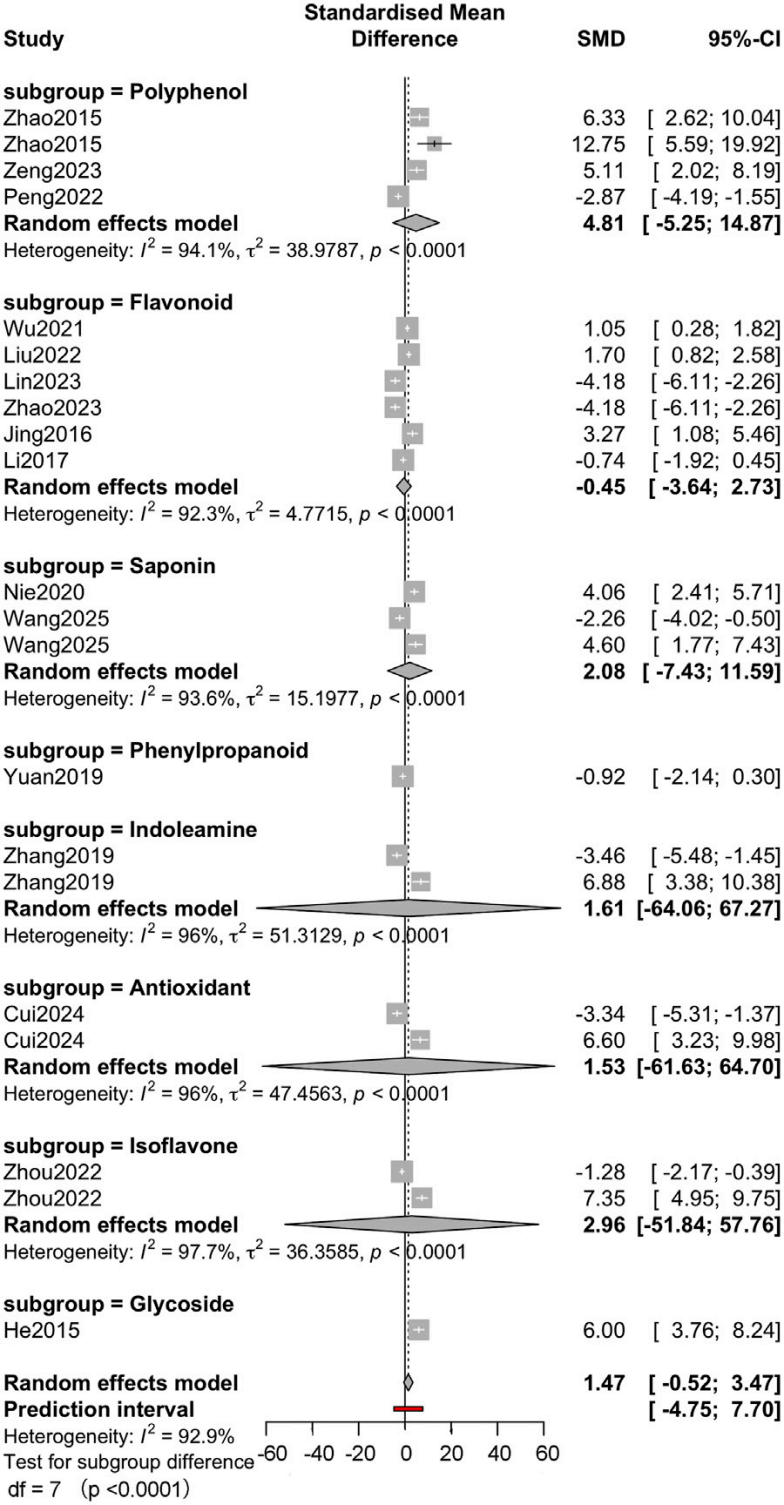
The molecular category subgroup analysis Random-effects subgroup meta-analyses by molecular class (polyphenols, flavonoids, saponins, antioxidants, glycosides, others). Subgroup p-value indicates test for subgroup differences.

**FIGURE 5:**
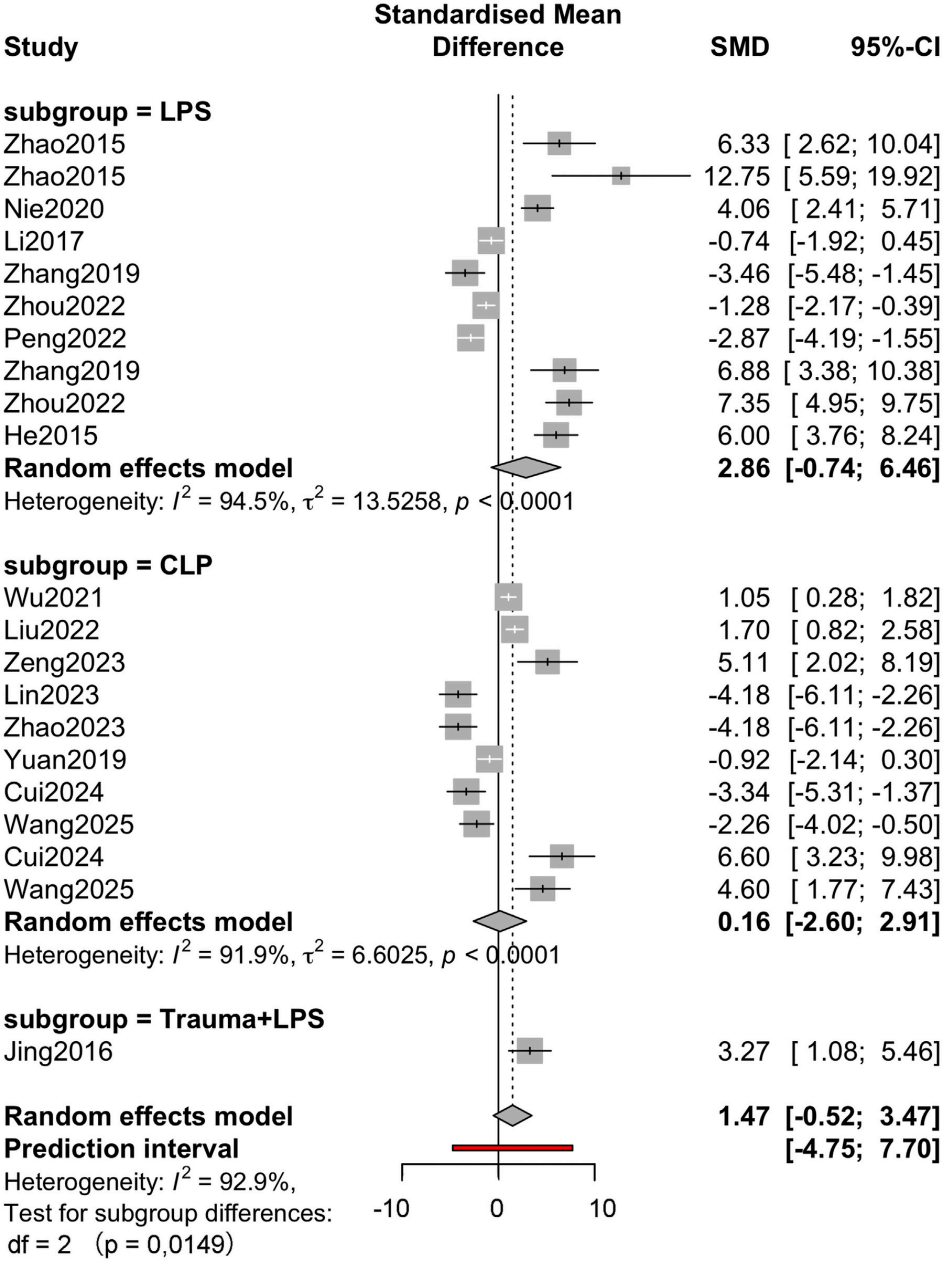
Subgroup analysis of sepsis modeling analysis Forest plot with subgroup-specific pooled estimates (SMD, 95% CI), I^2^ within subgroup and counts (k).

**FIGURE 6:**
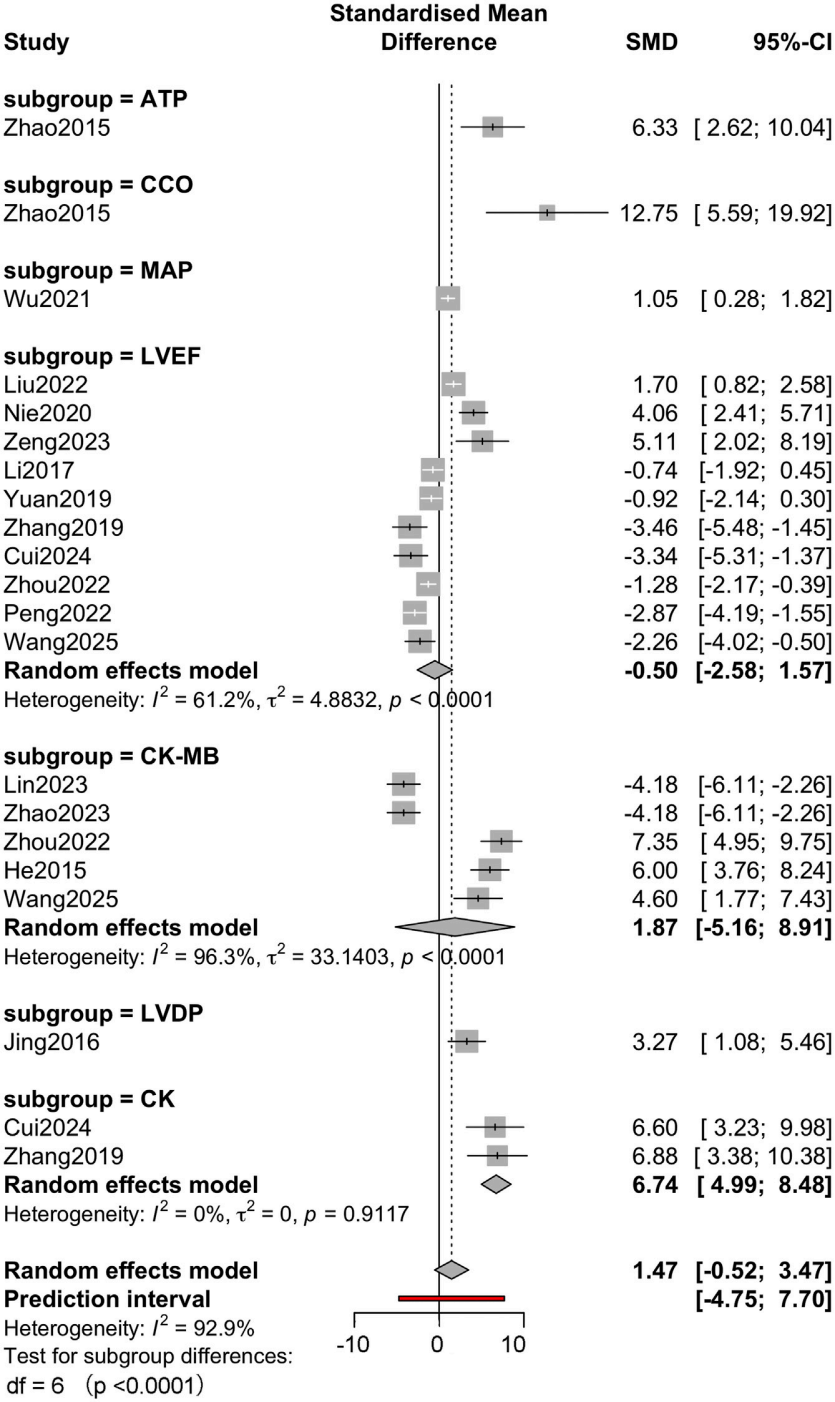
Analysis of outcome measures.

Subgroup analysis was performed according to molecular classification, sepsis modelling method and outcome measure to further explore the source of heterogeneity. The molecular category (Figure 4) subgroup analysis showed that the effect size varied. The effect size for polyphenols was large but imprecise (g = 4.81, 95% CI: −5.25–14.87), whereas the effect for flavonoids was relatively neutral (g = −0.45, 95% CI: −3.64–2.73). Subgroup heterogeneity remained significant within each subgroup (I^2^ > 90%), suggesting that the molecular class could not fully explain the variability. Subgroup analysis of sepsis modelling approaches (Figure 5) showed differences in effect estimates. The LPS-induced sepsis model showed a medium effect size (g = 2.86, 95% CI: −0.74–6.46), while the CLP-induced model subgroup had a smaller effect size (g = 0.16, 95% CI: −2.60–2.91). Only 1 study used the trauma + LPS model, with an effect size of 3.27 (95% CI: 1.08–5.46). There were no statistically significant differences between subgroups (p = 0.449). The analysis of outcome measures (Figure 6) showed significant differences between specific outcomes. Notably, outcomes related to myocardial biochemical markers, such as CK and CK-MB, had higher and more precise effect sizes compared with functional measures such as LVEF and MAP. For example, the CK result showed a robust effect (g = 6.74, 95% CI: 4.99–8.48), while the LVEF result showed a small and imprecise negative effect (g = −0.50, 95% CI: −2.58–1.57). The overall test of subgroup differences was statistically significant (p < 0.0001).

#### 3.4.3 Publication bias

**FIGURE 7:**
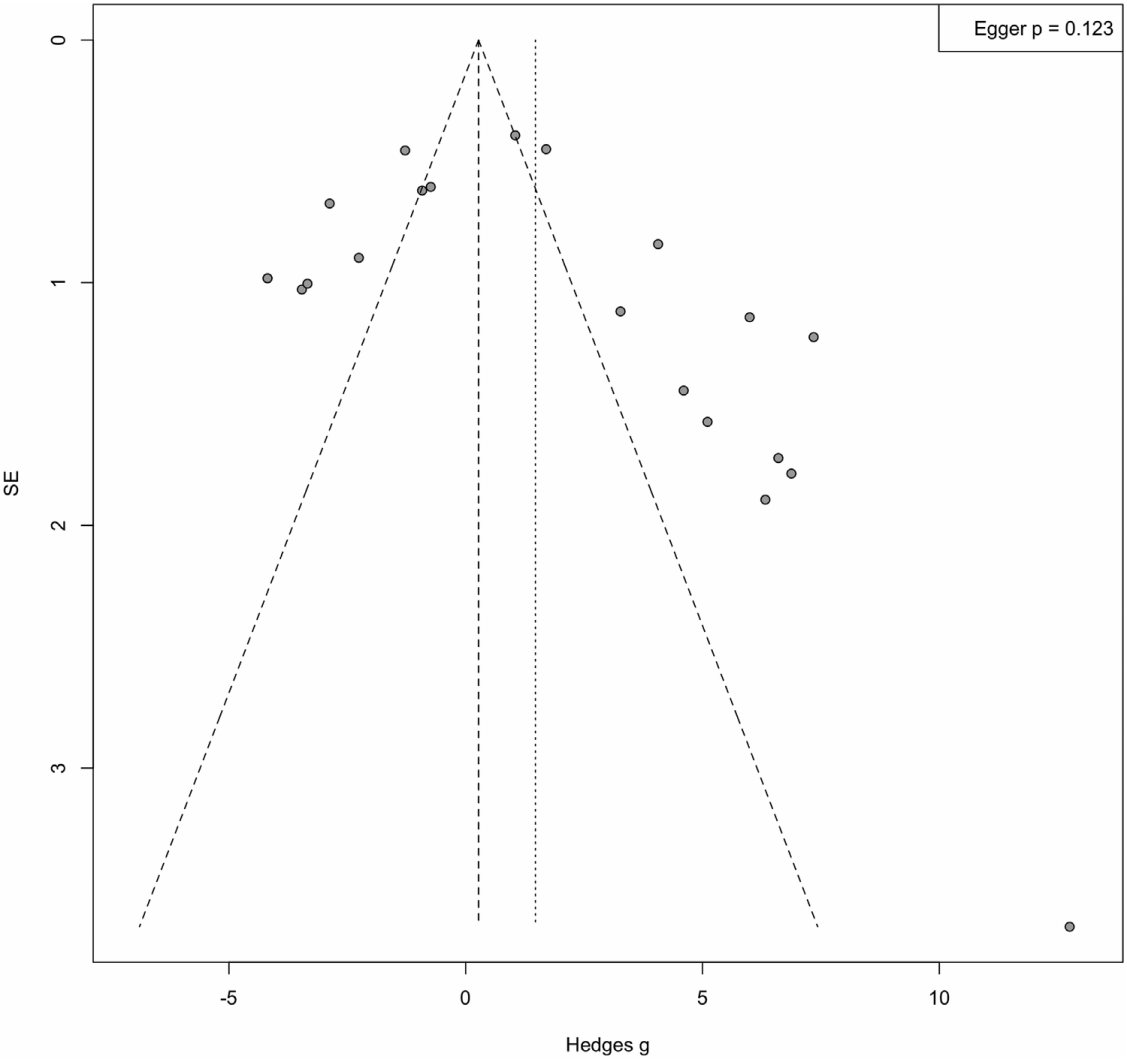
Funnel Plot and Egger’s regression. Funnel plot for assessment of small-study effects; Egger’s regression test p-value reported. Visual asymmetry should be interpreted cautiously given high heterogeneity.

Publication bias was assessed using funnel plots and Egger regression tests (Figure 7). Visual inspection of the funnel plots showed slight asymmetry, suggesting the possibility of publication bias. However, the Egger regression test did not show statistically significant asymmetry (p = 0.123). Given the large heterogeneity of the studies and the limited number of studies included, the interpretation of potential publication bias should be carefully considered.

#### 3.4.4 Sensitivity analysis

**FIGURE 8:**
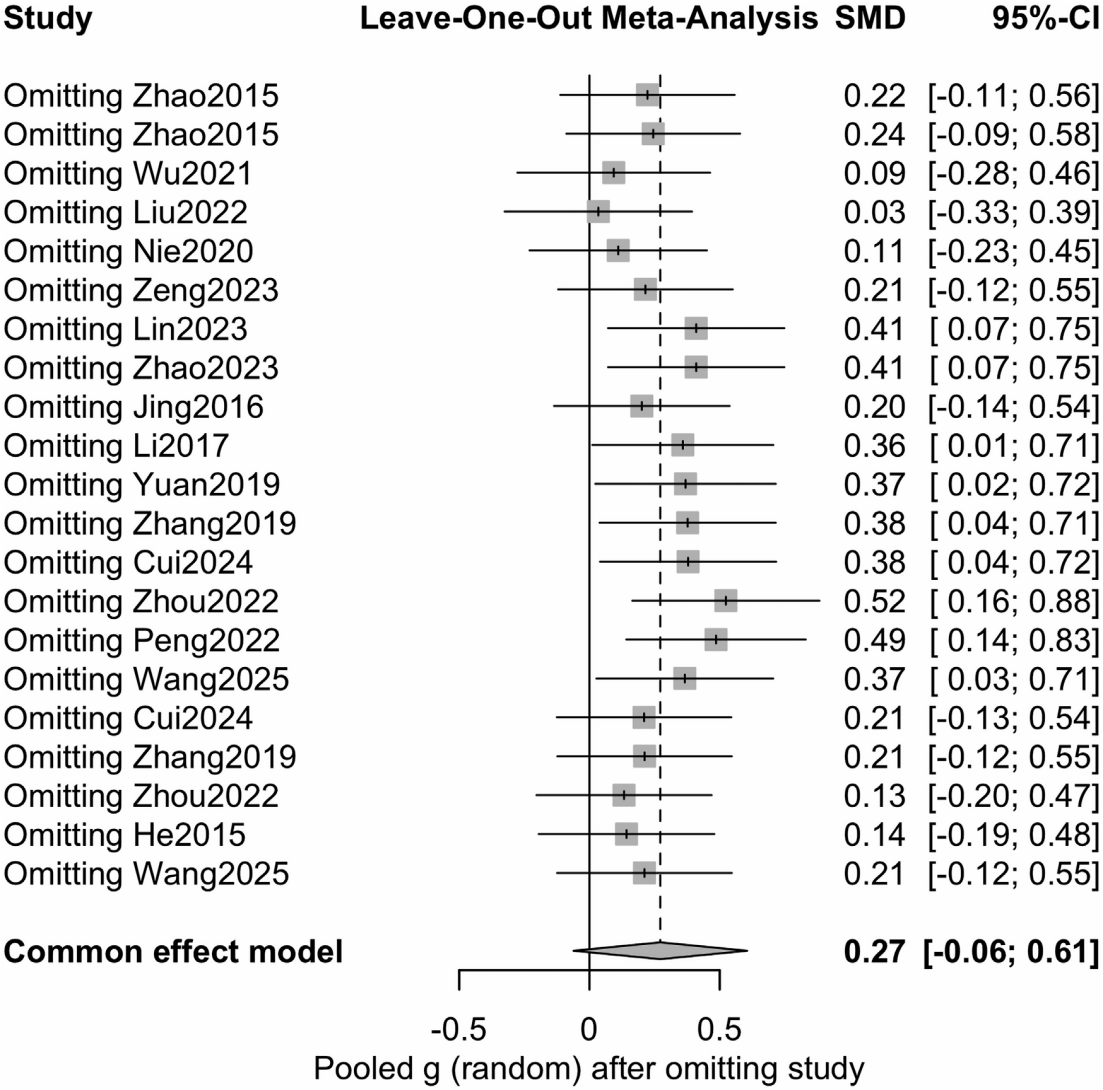
Leave-one-out sensitivity analysis.

The robustness of the overall pooled effect was assessed using a leave-one-out sensitivity analysis (Figure 8). Excluding any one study did not significantly change the pooled SMD, with effect sizes ranging from 0.03 to 0.52 in different iterations. These results support the robustness and stability of the conclusions of the primary meta-analysis.

In summary, the quantitative meta-analysis showed that active small molecules had generally positive but imprecise efficacy in animal models of abdominal trauma or sepsis-induced cardiomyopathy, with high statistical heterogeneity. Subgroup analyses suggested that molecular classification, sepsis model and specific outcome measures partially explained the observed heterogeneity.

## 4 Discussion

In this systematic review and meta-analysis, we comprehensively evaluated the protective effects of various active small molecules on SCM induced by abdominal trauma or sepsis in animal models. We included 17 studies covering multiple molecular classes and animal models, and performed quantitative analysis, which showed that SCM had an overall positive effect. Specifically, the pooled SMD for cardiac function and injury outcomes was 1.47 (95% CI: −0.52–3.47). Although the pooled effect suggested a potential cardioprotective effect, the wide confidence interval and high heterogeneity (I^2^ = 92.9%) highlighted significant differences in the strength and consistency of these effects among the included studies.

The potential protective mechanisms of active small molecules against SCM involve multiple biological pathways, particularly reducing inflammation, alleviating oxidative stress, protecting mitochondrial function and, subsequently, improving myocardial injury markers and cardiac function parameters. In subgroup analyses, polyphenols showed a significant but imprecise protective effect (SMD = 4.81), while the results for flavonoids were more ambiguous (SMD = −0.45). These differential findings suggest the existence of different molecular mechanisms, bioavailability, pharmacokinetic or dose-related factors that may explain the differences between the categories.

Previous preclinical and clinical studies have highlighted the pleiotropic effects of polyphenolic compounds such as resveratrol and rosmarinic acid, especially their potent antioxidant and anti-inflammatory activities. Zhao et al. reported that resveratrol significantly increased myocardial ATP and mitochondrial CCO activity in a rat LPS-induced sepsis model, highlighting mitochondrial protection as a key mechanism of cardioprotection (Zhao et al., 2015). These results are consistent with existing evidence from other cardiovascular studies that attribute the efficacy of polyphenols to antioxidant pathways targeting mitochondria, and the subsequent attenuation of oxidative stress-induced myocardial dysfunction (Su et al., 2024; Xu et al., 2020; Yang et al., 2024).

In contrast, the results for flavonoids in this meta-analysis were inconsistent. Although some individual flavonoids, such as luteolin and apigenin, showed moderate efficacy in specific cardiac outcomes, the overall results for flavonoids were relatively neutral. This finding is in stark contrast to previous studies that have highlighted the potent anti-inflammatory properties of flavonoids in various inflammatory disease models (Hu et al., 2015; Wu et al., 2023a; Wang et al., 2023). These variances may reflect differences in the severity of SCM animal models, differences in dosing regimens or inherent bioavailability limitations of flavonoids.

Subgroup analyses based on the method of sepsis induction further demonstrated that the efficacy of active small molecules varied across models. The LPS-induced endotoxemia model showed a greater protective effect (SMD = 2.86) than the CLP model (SMD = 0.16). This difference may stem from the pathophysiological differences between endotoxemia (acute inflammatory stimulation, systemic cytokine surge) and CLP-induced polymicrobial sepsis, which is closer to the clinical sepsis state (Wang et al., 2021; Rosado-Franco et al., 2021; Yuan et al., 2022). Therefore, molecules with acute anti-inflammatory effects, such as resveratrol and rosmarinic acid, may show higher efficacy in the LPS model, reflecting their rapid modulation of cytokine profiles and acute inflammation, but their sustained effects may be weaker in the more complex and prolonged CLP-induced sepsis environment.

Further analysis based on outcome measures found significant differences between biochemical markers (CK, CK-MB) and functional cardiac parameters (LVEF, MAP). Biochemical indicators such as CK-MB showed sustained protective effects, while functional indicators such as LVEF showed mixed and unclear efficacy. This observation is consistent with existing literature suggesting that biochemical markers are sensitive indicators of early myocardial injury and recovery, whereas echocardiographic parameters such as LVEF may reflect more complex, multifactorial changes in myocardial function and haemodynamics, which evolve over time (Wang et al., 2022; Wu et al., 2023b; Sun et al., 2024).

Comparison with previous literature: Previous systematic reviews have highlighted the potent antioxidant and mitochondrial protective properties of polyphenols, especially resveratrol, which are well aligned with our current findings (Zeng et al., 2023; Taha et al., 2023). Similarly, flavonoids, despite their variable overall efficacy in our analysis, have been widely reported to exhibit cardioprotective properties through anti-inflammatory and antioxidant mechanisms, although results have been inconsistent across experimental contexts (Huajie, 2015).

Septic cardiomyopathy remains an important unmet clinical need. Current clinical management is primarily supportive (including careful fluid resuscitation, vasopressors and inotropes when indicated), and there are no widely accepted, evidence-based pharmacologic agents that specifically prevent or reverse sepsis-induced myocardial dysfunction (L'Heureux et al., 2020). Recent preclinical data indicate that certain small molecules (notably, polyphenolic compounds such as resveratrol and related agents) can modulate key pathophysiologic pathways implicated in SCM, including pro-inflammatory signalling, oxidative stress and mitochondrial dysfunction, and thereby protect cardiac tissue in animal models. However, translating these findings to human therapy faces several practical barriers: many polyphenols and small molecules exhibit poor oral bioavailability and limited tissue penetration; robust safety/toxicity and PharmacoKinetics and PharmacoDynamics (PK/PD) characterisation in relevant models is often lacking (Di Lorenzo et al., 2021); dosing regimens and timing relative to sepsis induction require careful definition; and functional cardiac endpoints (rather than biochemical markers only) must be demonstrated in reproducible, cross-species studies. To facilitate translation, we propose a staged roadmap: (1) standardised, high-quality preclinical studies that adhere to ARRIVE recommendations and include PK/PD and formal toxicity arms; (2) the demonstration of reproducibility across species and in more clinically relevant models (e.g., CLP and larger animal models, where feasible); and (3) early-phase clinical studies that prioritise safety and objective cardiac functional endpoints (e.g., echocardiographic strain measures, haemodynamic indices and clinically relevant outcomes) (Percie du Sert et al., 2020; Nicol et al., 2021). These steps aim to bridge promising preclinical signals and rigorous human evaluation.

Lipopolysaccharide-induced endotoxemia and CLP-induced polymicrobial sepsis differ markedly in terms of their kinetics of cytokine responses, bacterial dissemination and clinical course; consequently, therapeutic efficacy observed in LPS models may not translate to CLP models or to human sepsis cases. Previous comparative studies have documented these kinetic and immunologic differences (Remick et al., 2020; Seemann et al., 2017) accordingly, we suggest prioritising CLP models for translational studies, while recognising the value of LPS models for testing acute anti-inflammatory effects. Importantly, our review focused on abdominal-origin models (including abdominal trauma and abdominal-source sepsis); therefore, the findings may not be generalisable to sepsis arising from other sources (e.g., pulmonary or urinary tract infections), and it is possible that the inclusion of studies focused on other sepsis origins could yield different pooled estimates due to source-specific pathophysiology.

Notably, although previous studies have frequently documented significant protective effects of antioxidants such as vitamin C, our meta-analysis included only limited data (Cui et al., 2024), highlighting the need for further exploration and the validation of such interventions in animal SCM models. Similarly, the favourable effects observed for saponins (astragaloside IV, ginsenoside Rc) are consistent with previous findings documenting anti-inflammatory, anti-apoptotic and mitochondrial protective properties in other experimental models of myocardial injury.

Our meta-analysis has several strengths. First, to our knowledge, this is the first systematic, comprehensive study of active small molecules in animal models of abdominal trauma/sepsis-induced cardiomyopathy. In addition, we used a rigorous and comprehensive approach, including extensive subgroup analyses, sensitivity testing and systematic risk of bias assessment, using the validated SYRCLE tool.

Several important limitations should, however, temper the interpretation of our findings. First, substantial heterogeneity persisted across studies (I^2^ = 92.9%), which likely reflects multiple sources of clinical and methodological variability, that is, inconsistent reporting of dosing regimens and timing relative to sepsis induction, heterogeneity in sepsis models and model severity, differences in animal species/strains, as well as variable definitions and timing of the outcome measurements. Second, many of the included studies incompletely reported key experimental design features, increasing the risk of selection and detection bias. Moreover, these methodological shortcomings not only threaten internal validity by inflating effect sizes but also reduce the reproducibility and external generalisability of the findings. Third, the modest number of studies overall, combined with sparse and inconsistent reporting of quantitative dosing, route and timing, limits the feasibility and reliability of meta-regression or stratified analyses. Fourth, the available literature placed greater emphasis on biochemical markers than on standardised functional cardiac outcomes; biomarker improvements may not equate to meaningful or durable functional recovery. Fifth, adverse effects and formal toxicity assessments were generally not reported, restricting any assessment of benefit/risk for the compounds studied. Sixth, small-study effects and possible publication bias cannot be excluded, and this may have led to the overestimation of efficacy. Seventh, many studies lacked pre-registration or publicly available raw data, limiting transparency and reproducibility. Finally, several included studies originated from a limited number of research groups or geographic regions, which may have reduced methodological diversity and increased the risk of confirmation bias. Future work should follow ARRIVE recommendations, include PK/PD and toxicity arms, pre-register experimental protocols, use harmonised endpoints, and make raw datasets available to enable reliable synthesis.

Our findings highlight the potential of active small molecules, especially polyphenols, as preventive treatments for SCM and highlight their mechanisms of inflammation reduction, oxidative stress reduction and mitochondrial protection. However, the large differences observed suggest that further standardised, rigorously designed preclinical trials are urgently needed. Future studies should prioritise methodological rigour, including detailed reporting of randomisation, allocation concealment and blinding, as well as systematic investigation of dose-response relationships, timing and long-term cardiac outcomes.

## 5 Conclusion

In summary, this meta-analysis demonstrates that active small molecules have generally positive but heterogeneous protective effects against abdominal trauma or sepsis-induced cardiomyopathy in animal models. Polyphenols showed particularly promising cardioprotective effects through antioxidant and mitochondrial protective mechanisms, although large variability remained. Future studies should prioritise methodological rigour, improved standardisation and translation-oriented studies to validate these promising preclinical findings and guide clinical application.

## Data Availability

The original contributions presented in the study are included in the article/supplementary material, further inquiries can be directed to the corresponding authors.
